# The Characteristics of Carbon, Nitrogen and Sulfur Transformation During Cattle Manure Composting—Based on Different Aeration Strategies

**DOI:** 10.3390/ijerph16203930

**Published:** 2019-10-16

**Authors:** Yue Wang, Shanjiang Liu, Wentao Xue, He Guo, Xinrong Li, Guoyuan Zou, Tongke Zhao, Hongmin Dong

**Affiliations:** 1 Institute of Plant Nutrition and Resources, Beijing Academy of Agricultural and Forestry Sciences, Beijing 100087, China; yuewang2008@126.com (Y.W.);; 2 Urban Construction School, Beijing City University, Beijing 100083, China; 3 Department of Environmental and Occupational Health Sciences, University of Washington, Seattle, WA 98105, USA; 4 Institute of Environment and Sustainable Development in Agriculture, Chinese Academy of Agricultural Sciences, Beijing 100081, China

**Keywords:** compost, aeration, gas emission, mass balance, carbon, nitrogen, sulfur

## Abstract

This study aimed to investigate the characteristics of gaseous emission (methane—CH_4_, carbon dioxide—CO_2_, nitrous oxide—N_2_O, nitric oxide—NO, hydrogen sulfide—H_2_S and sulfur dioxide—SO_2_) and the conservation of carbon (C), nitrogen (N), and sulfur (S) during cattle manure composting under different aeration strategies. Three aeration strategies were set as C60, C100, and I60, representing the different combinations of aeration method (continuous—C or intermittent—I) and aeration rate (60 or 100 L·min^−1^·m^−3^). Results showed that C, N, S mass was reduced by 48.8–53.1%, 29.8–35.9% and 19.6–21.9%, respectively, after the composing process. Among the three strategies, the intermittent aeration treatment I60 obtained the highest N_2_O emissions, resulting in the highest N loss and greenhouse gas (GHG) emissions when the GHG emissions from power consumption were not considered. Within two continuous aeration treatments, lower aeration rates in C60 caused lower CO_2_, N_2_O, NO, and SO_2_ emissions but higher CH_4_ emissions than those from C100. Meanwhile, C and N losses were also lowest in the C60 treatment. H_2_S emission was not detected because of the more alkaline pH of the compost material. Thus, C60 can be recommended for cattle manure composting because of its nutrient conservation and mitigation of major gas and GHG emissions.

## 1. Introduction

The rapid development of China’s livestock industry has increased the generation of animal manure. Livestock manure in China has reached an annual volume of 3.8 billion tons [1]. Approximately 40% of manure has not been used properly because of the long-term separation of animal breeding and manure land application, which results in severe pollution and a significant loss of nutrient resources [1]. It’s estimated that the nutrient losses have reached 3 million tons and greenhouse gas (GHG) emissions have exceeded 100 million tons of CO_2_ equivalent (CO_2_eq) per year because of unused manure and other organic agriculture waste [2,3]; and odor emanation also has resulted in numerous complaints. Pollution prevention of livestock and poultry breeding has been a focus of the current agricultural work in China. Among the different manure treatments, composting is used as an alternative to traditional manure management and is widely adopted by the animal production industry in China. Composting can transform complex organic substrates into sanitary and stabilized end products, which serve as organic fertilizer that can improve soil fertility and be used for crop production [4,5].

During composting, manure undergoes biological degradation that results in gaseous products, such as methane (CH_4_), carbon dioxide (CO_2_), nitrous oxide (N_2_O), nitric oxide (NO), hydrogen sulfide (H_2_S), and sulfur dioxide (SO_2_). Among these gases, CH_4_ and N_2_O are identified as two major non-CO_2_ GHGs which have strong global warming potentials (GWP), being 28 and 265 times higher than that of CO_2_, respectively [6]. CO_2_ is also an important GHG, although the CO_2_ emissions from agriculture are not estimated because annual net CO_2_ emissions are assumed to be zero—the CO_2_ photosynthesized by plants is returned to the atmosphere as respired CO_2_ [7,8]. Considering the air pollution issue, NO and SO_2_ are criteria pollutants defined in China Air Pollution Prevention Action Plan. NO and SO_2_ participate in the regulation of oxidant balance in the atmosphere; meanwhile, SO_2_ is the precursor of acid rain and NO may contribute to acid rain formation, photochemical smog generation, and ozone depletion [9,10]. H_2_S is an important malodor that can reduce the wellbeing of workers and impact the life of residents. These gas emissions create environmental concerns and reduce the agronomic value of compost as a soil amendment. With the rapid development of the manure composting industry, performing research to quantify the emissions of different gases and determining the available amount of nutrients that can be used as soil amendments and organic fertilizers is of high importance.

Aeration is an important factor that influences composting. Proper aeration can help accelerate composting, improve the quality of compost products, and reduce energy consumption. Excessively high aeration rate may promote heat dissipation, thus causing low temperature, which is unsuitable for sanitary standards. Meanwhile, high aeration rate results in large losses of compost nutrient elements and high energy costs [11,12]. However, if the aeration is not enough, it also results in low temperature because the oxygen (O_2_) supplied for the metabolism of aerobic microorganisms is insufficient. Moreover, anaerobic condition would occur in the compost pile, causing large emissions of malodor gases and CH_4_ [13,14,15]. Shen et al. [13] applied aeration rates of 10, 100, and 200 L·min^−1^·m^−3^ for pig manure composting, and found that the aeration rate should be higher than 100 L·min^−1^·m^−3^ to achieve successful composting because the compost temperature at the lowest aeration rate did not exceed 40 °C. After a comprehensive evaluation of compost effect and energy cost, Guo [16] indicated that the aeration rate should be approximately 100 L·min^−1^·m^−3^ for co-composting of pig manure and pig carcass during summer and 60 L·min^−1^·m^−3^ during winter. Jiang et al. [12] compared CH_4_, N_2_O, and NH_3_ emissions from pig manure compost with different aeration rates (39.3–117.9 L·min^−1^·m^−3^) and different aeration methods (continuous or intermittent). High aeration rate resulted in high N_2_O and NH_3_ emissions, low CH_4_ emissions, and large total nitrogen (TN) losses. Meanwhile, intermittent aeration released high N_2_O emissions and low NH_3_ emissions [12]. For S-related gas emissions, volatile sulfur compound (VSC) emissions were high with low aeration rate [15,17].

However, no studies have investigated SO_2_ emissions during composting, and limited literature has reported NO emissions during composting [9,18,19]. SO_2_ can be an incomplete oxidation product with the function of microorganisms. It’s reported that there might be certain SO_2_ release and concentration at locations with large quantities of manure storage, like animal barns [20]. Elliott and Travis [21] reported the chemical combinations such as CO_2_, H_2_S, and SO_2_ can form carbonyl sulfide (COS), which plays an important role in the global sulfur cycle and is relevant for climate change due to its role as a greenhouse gas, in aerosol formation and atmospheric chemistry [22]. It has been reported that COS can be detected in the headspace above feedlot compost [21]. Meanwhile, composting increased the atmospheric concentration of NO [9]. NO usually occurred simultaneously with N_2_O emission [19,23]. The higher concentrations of nitrite in the composting samples enhanced the NO emission [19]; however, no such significant relationship was observed in Tsutsui et al. [23]. Hao and Chang [9] reported the concentration of NO for the active aeration treatment was lower at the beginning and higher towards the end of composting compared with that for the passive aeration treatment. Martin and Dewes [18] reported that NO_X_-N loss was lower than 5% during manure composting. Although the NO emission would be low, we cannot ignore it since it may contribute to acid rain formation and other environmental issues [9]. Considering that NO is a product of nitrification and denitrification of composting pile, the simultaneous monitoring of N_2_O and NO can help to reveal the mechanism of nitrification and denitrification during composting.

Although several researches have studied the effects of aeration on gas emissions or nutrient conservation, they have focused on the common subjects such as CH_4_, CO_2_ and N_2_O, with limited data being available for the simultaneous evaluation of six major polluting gases (i.e., CH_4_, CO_2_, N_2_O, NO, H_2_S, SO_2_) during manure composting. Therefore, this study aimed to investigate the emission characteristics of CH_4_, CO_2_, N_2_O, NO, H_2_S, and SO_2_, as well as elucidate the interactions and transformations between the gas components, compost pile components, and the environmental conditions under different aeration strategies. Aeration rate was set in an appropriate range to achieve successful composting, making the results valuable based on the necessity of practical application. The results would be helpful to understand the gas emission characteristics from compost process, and serve as guide in finding an aeration strategy with low GHG or major gas emissions and maintaining large nutrient elements in the final compost products.

## 2. Materials and Methods

### 2.1. Raw Material and Composting Preparation

Cattle manure was obtained from a beef cattle fattening farm in Beijing. The beef cattle raised in the farm were managed on a bedding system, with fresh manure excreted on the chopped corn stalk bedding material and stored for approximately three to four months in the bedding house before being cleaned out for composting. The use of aged beef cattle manure as raw material for composting is common for both beef cattle bedding system and beef cattle feedlot system [9,24]. For composting, cattle manure was thoroughly mixed with some chopped wheat straw (<20 mm length) as a bulking agent at a ratio of 6:1 (fresh weight). The added straw caused the pile mixture to be dry, thus almost 1.6 L fresh water was added to adjust the initial moisture content (MC) of the compost pile to 65% for each compost reactor. Table 1 lists the characteristics of the raw and mixed materials. The C/N ratio of compost mixture was 14.2 ± 0.26, which was comparable with the ratio used in Zeng et al. [25] and Zhou [26], being as 13.20–14.57. Although the C/N ratio was low, it was representative of that used in the Chinese composting plant, as achieving high initial C/N ratio usually burdened farmers with extra costs for additional straw [12,26].

The composting experiment was conducted using a modified composting reactor system from Zang et al. [27]. The composting vessel was made of stainless steel with an effective volume of approximately 60 L (inner diameter, 0.36 m; height, 0.6 m). About 30.8 kg initial compost mixture was added in each reactor, with the initial working volume of 50 L, leading to an initial density of 616 kg·m^3^. The outer wall of the box was insulated with 0.05 m rock mineral cotton between two stainless steel layers to retain the heat generated during composting. The lid of each vessel had two outlets to allow a temperature sensor to be inserted and for gas within the vessel to be sampled. At the bottom of each reactor, a sieve plate with 5 mm openings was installed to support the composting bed and ensure the air being pumped in were uniformly distributed. There were two ports on the lateral lower part of each reactor; one for leachate collection and the other for air input. However, no leachate was observed in the experiment. Air was provided by an air pump, and the flow rates to each reactor could be controlled with rotor flow meters.

In this study, three aeration strategies were applied: continuous aeration with a rate of 100 L·min^−1^·m^−3^ (C100), continuous aeration with a rate of 60 L·min^−1^·m^−3^ (C60), and intermittent aeration with an average aeration rate of 60 L·min^−1^·m^−3^ (I60, 10 min on with a rate of 180 L·min^−1^·m^−3^, 20 min off) according to the studies of Guo [16] and Jiang et al. [12] to simulate the aeration at relatively low and high level. Each treatment was conducted in triplicate. The compost temperature in each reactor was recorded hourly with a HOBO temperature meter (U23-002, Onset Inc., City, State abbreviation, USA) placed 10 cm below the compost surface. Ambient air temperature in the laboratory was recorded hourly. The composting period lasted 31 days, from 6 September 2018 to 8 October 2018.

### 2.2. Sampling and Analysis

#### 2.2.1. Gas Sampling and Analysis System

**Figure 1:**
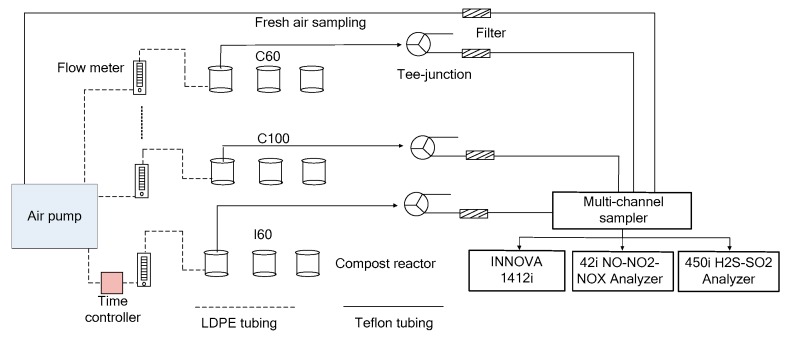
Schematic of the experimental setup. C60—continuous aeration with a rate of 60 L·min^−1^·m^−3^, C100—continuous aeration with a rate of 100 L·min^−1^·m^−3^, I—intermittent aeration with an average rate of 60 L·min^−1^·m^−3^.

**Figure 2:**
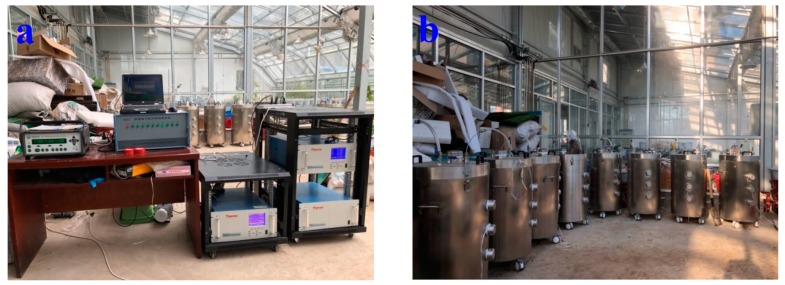
A photographical view of the experimental setup. (**a**) the instruments, (**b**) composting reactor.

Gas sampling and analysis were performed using a modified gas monitoring system from Wang et al. [28]. A photoacoustic multigas analyzer (Innova 1412i, LumaSense Technologies, Ballerup, Denmark), a NO analyzer (model 42i, ThermoFisher Scientific Inc., Waltham, MA, USA), a H_2_S–SO_2_ analyzer (model 450i, ThermoFisher Scientific Inc., Waltham, MA, USA) along with a multichannel sampler (fabricated at the Institute of Environment and Sustainable Development in Agriculture, Chinese Academy of Agricultural Sciences, Beijing, China) were used to sample and analyze air samples from nine composting reactor air sample outlets and one fresh air supply. Before the measurements, the gas analyzers were checked and calibrated as required using individual CH_4_, CO_2_, N_2_O, NO, SO_2_, H_2_S, and nitrogen (N_2_) standard calibration gases procured from the National Standard Material Center in Beijing, China. During monitoring, the air samples for the nine air outlets on the lids of each reactor and the fresh air inlet was pumped into the multi-channel sampler by turns, and then analyzed for 10 1-min cycles with NO analyzer and H_2_S–SO_2_ analyzer, whereas five 2-min cycles were used for the photoacoustic multigas analyzer (CH_4_, CO_2_, N_2_O) simultaneously. The difference in sample analysis cycle time between the two types of instruments was due to their different response times, with the Innova multigas analyzer taking a longer time (2 min) to stabilize and get one reading, while the NO analyzer and H_2_S–SO_2_ analyzer both taking 1 min to stabilize and get one reading. Readings associated with the last sample for each analyzer were taken for the measured values, and previous cycles were used to stabilize the readings. Thus, it took a total of 100 min to complete one system sampling cycle. Each analyzer ran for 24 h every day to obtain real-time monitoring results. A total of 13–14 measurements were made per day for each reactor during the entire monitoring period. A schematic and photographs of the experimental setup are shown in Figure 1 and Figure 2.

#### 2.2.2. Determination of Gaseous Emission Flux

Concentrations of CH_4_, CO_2_, N_2_O, NO, SO_2_, and H_2_S gases of fresh and exhaust air were automatically measured and recorded. With a known aeration rate, the gaseous emission fluxes from the compost reactors were calculated using the following equation [29]:

(1) $$ER_{i}=\left(C_{out\_i}-C_{in\_i}\right)\times VR\times 0.001\times 60\times 24$$

where $ER_{i}$ is the mean emission flux on day i (mg·m^−3^·d^−1^), $C_{in\_i}$ is the gas concentration of inlet air on day i (mg·m^−3^), $C_{\mathrm{out}\_i}$ is the gas concentration of outlet air on day i (mg·m^−3^), VR is the aeration rate (L·min^−1^·m^−3^), 0.001 is the conversion factor from L to m^3^, 60 is the conversion factor from min to hour, and 24 is the conversion factor from hour to day.

#### 2.2.3. Compost Sample Collection and Analysis

During composting, 200 g homogeneous compost samples were taken after 0, 6, 13, 20, and 31 d. The samples were divided into two; one part was stored at 4 °C until analysis, and the other part was air dried, powdered in an agate mortar, passed through a 0.1 mm sieve, and thoroughly mixed for analysis. Fresh samples were evaluated in terms of pH, MC, ammoniacal nitrogen (NH_4_^+^-N), and nitrate nitrogen (NO_3_-N) levels, dissolved organic carbon (DOC), and seed germination index (GI), and air-dried samples were assessed in terms of organic matter (OM), total organic carbon (TOC), TN, and total sulfur (TS).

MC was determined by drying the fresh samples at 105 °C to a constant weight. TS was measured using an elemental analyzer (Vario, MACRO cube, Hanau, Germany). OM, TOC and TN contents were measured based on the Chinese national standard of organic fertilizer (NY 525-2012) [30]. pH was measured using a pH meter (MP521, Shanghai, China) with 1:10 aqueous extract (w/v). To determine DOC, fresh samples were extracted at 25 °C with ultrapure water at a ratio of 1:5 (w/w) using a shaker at 200 rpm for 24 h. This was followed by centrifugation at 8000 rpm for 5 min and filtration with a 0.45 μm nylon syringe filter and then detected using an automated TOC analyzer (TOC-V, Shimadzu, Kyoto, Japan). NH_4_^+^-N, and NO_3_^−^-N were extracted with 50 ml 2 mol·L^−1^ KCl extracts [solid: extractant, 1:10 (w/v)] and analyzed using a segmented flow analyzer (FIAstar 5000, Foss, Hillerød, Denmark) [31]. For analyzing GI, deionize water was added to the fresh compost samples to attain a 1:10 solid: water ratio (w/v) and the aqueous extracts were used as germination media. Aqueous extract (5 ml) was pipetted into petri dishes packed with a piece of filter paper. Ten Chinese cabbage seeds were evenly scattered on the filter paper and incubated at 25 ± 1 °C for 48 h in the dark. Deionized water was used as a control. After incubation, the number of germination seeds and the primary root length were measured and expressed as a percentage of the control as the germination index [5].

### 2.3. Statistical Analyses

The differences of C, N, S loss and GHG emissions between treatments were obtained through one-way ANOVA using Duncan’s LSD test and 95% confidence interval to compare the influence of the aeration treatment on the element losses and gas emissions. Pearson’s correlation coefficient was used for the analysis of bivariate correlations between the compost pile temperature and the CO_2_ emission to reveal the influence of temperature on gas emissions. SPSS 20.0 software (SPSS Inc., Chicago, IL, USA) was used for all statistical analyses.

## 3. Results and Discussions

### 3.1. Change of Temperature, MC and pH

**Figure 3:**
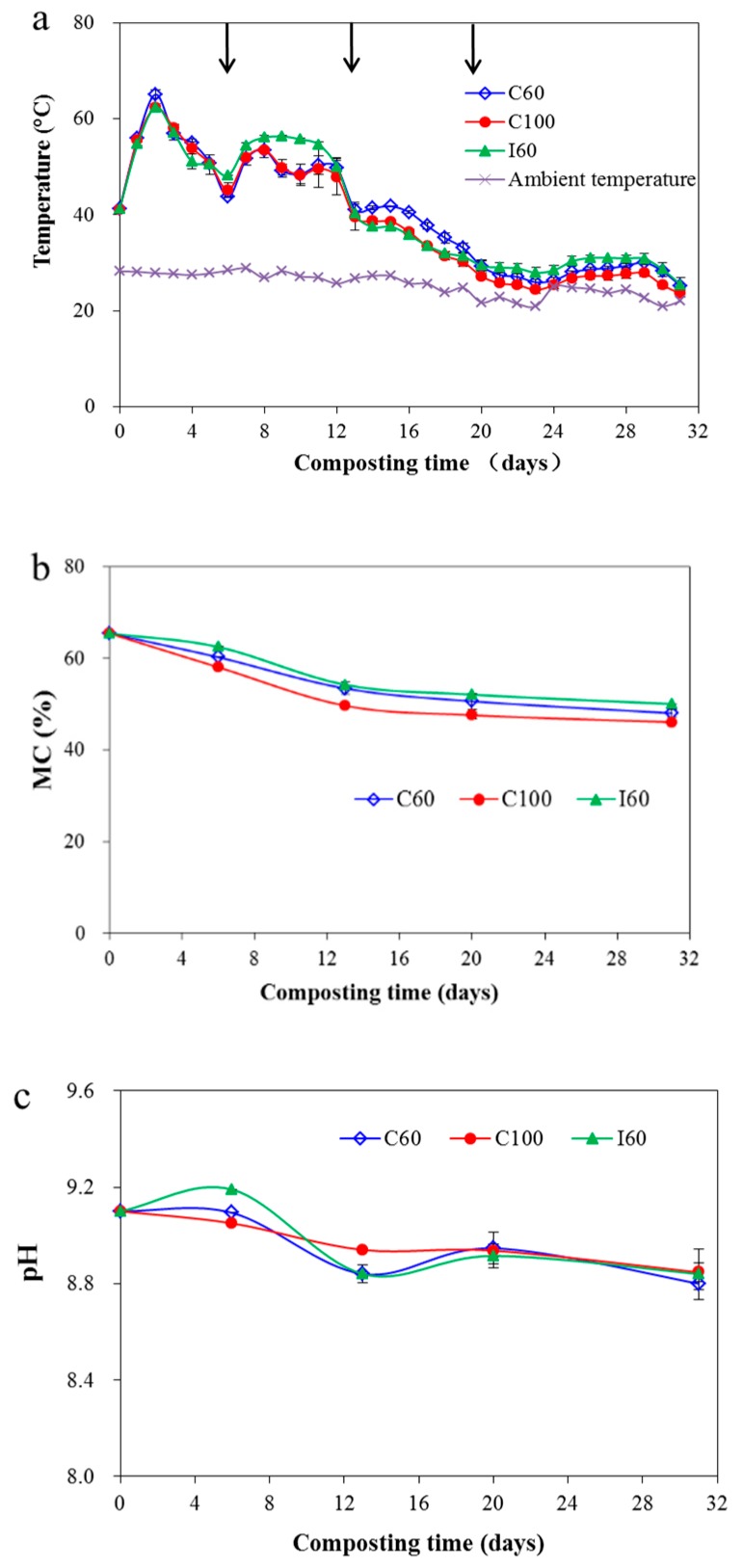
Changes of temperature (**a**), MC (**b**) and pH (**c**) during the composting of cattle manure. Results are the mean of three replicates and error bars indicate standard deviation. Arrows indicate the time of turning. For abbreviations see Figure 1.

During composting, the temperature of composting pile increased with the decomposition of available organic materials by indigenous microorganisms which produced energy [32]. Figure 3a showed that the temperature profiles of the three treatments were similar, with all treatments rapidly increasing and reaching the thermophilic phase (>50.0 °C) within two days. With the first turning on the sixth day, the temperatures of all three treatments rapidly decreased and increased to higher than 50 °C after the next day. For the temperature profiles of the periods between the first and second turns, the highest temperature was observed in the I60 group. Intermittent aeration achieved a relatively higher temperature than continuous aeration because it could preserve heat in the pile. Shen et al. [13] reported too much higher aeration rate would cause more quick decrease of pile temperature during manure composting, and thus shortened the thermophilic phase, which could be the reason for the lower temperature in C100 during the period between the first turn and the second turn in this study. For the two aeration methods with continuous or intermittent manner but with the same average rate, Yang et al. [33] found the continuous aeration led much lower pile temperature during the whole composting process when compared with the continuous aeration method, which was comparable with the results of this study. However, Jiang et al. [12] reported that under the same average aeration rate, the temperature under the intermittent aeration would decrease more quickly than that under the continuous one during the thermophilic phase, as the instant aeration rate caused higher moisture loss, and led the MC to be quickly reduced to lower than 40% which even inhibited microbial activity. However, the decrease of MC in this study was slow, and the MC was always kept higher than 50% which was suitable for microbial function, and thus didn’t lead to the reduction of pile temperature. The discrepancy between these studies could be due to differences in compost materials, experimental conditions, and moisture content [34]. The second turn on the 13th day did not retrieve the compost pile temperature, which may be caused by the depletion of easily degradable organic material such as DOC. The temperatures of the three treatments were maintained higher than 50 °C for more than seven days, satisfying the Chinese national standard NYT1168-2006 for composting [35]. Meanwhile, the final GI for C60, C100 and I60 were 84.4 ± 3.1%, 70.1 ± 2.3% and 80.4 ± 8.1%, respectively, all above the recommended GI value of 50% which meant the final compost was mature and was not inhibitory to plant growth [4,31]. However, it can be observed that C100 had the lowest GI among the three strategies. Bernal et al. [4] reported that the optimum temperature range for composting was 40–65 °C, and the always lower temperature in C100 during the composting period between the first turn and the third turn might be the reason for its final lower GI.

The thermophilic phase achieved in this trial was a little shorter than that in other compost systems, and the peak temperature was relatively lower [5,34]. These results might be attributed to two aspects. One could be the difference of beef cattle manure and other kinds of animal manure. Usually cellulose and lignin may account for 50% of dry matter (DM) content of cereal straws, which are the major feed for beef cattle [36], thus more cellulose and lignin are reserved in the beef cattle manure, whose degradation rates are limited during the composting period [37,38]. The other aspect might be less amount of readily biodegradable matter in the aged manure [38,39], which has been stored in the bedding house for nearly 3 months. Li et al. [39] compared the composting process of fresh dairy manure with that of aged dairy manure, and found that the peak temperature of the aged manure was 61.7 °C which was 7 °C lower than that of the fresh manure compost.

The MC decreased mainly during the first two weeks, with much water evaporated during the thermophilic phase (Figure 3b). The highest aeration rate in C100 achieved the lowest MC during the composting process. Li et al. [5] reported a similar pattern with MC being reduced from 68% to 55% during the first 30 days of pig manure composting, and the pile temperature was reduced to 35 °C accordingly during this period. While the final MC was reduced to lower than 35% with the compost period being extended to 90 days.

The pH of the compost pile in C60 and I60 showed a fluctuation pattern during the composting period (Figure 3c), similar to the fluctuation pattern observed by Wang et al. [34]. The initial pH increase in the compost was due to the ammonification and mineralization of organic nitrogen, which increased pH levels [40,41]. The following decrease in pH might be due to the formation of low-molecular-weight fatty acids, the release of H^+^ as a result of microbial nitrification by nitrifying bacteria, and the volatilization of NH_3_ [34,41,42]. Subsequently, the decomposition of organic acid and continuous CO_2_ emissions increased and decreased the pH in C60 and I60 treatments successively [34,41]. However, continuous high aeration in C100 treatment would expel high NH_3_ emissions outside of the pile, thus causing a continuous decrease of pH.

### 3.2. Evaluation of C Transformation during Composting

**Figure 4:**
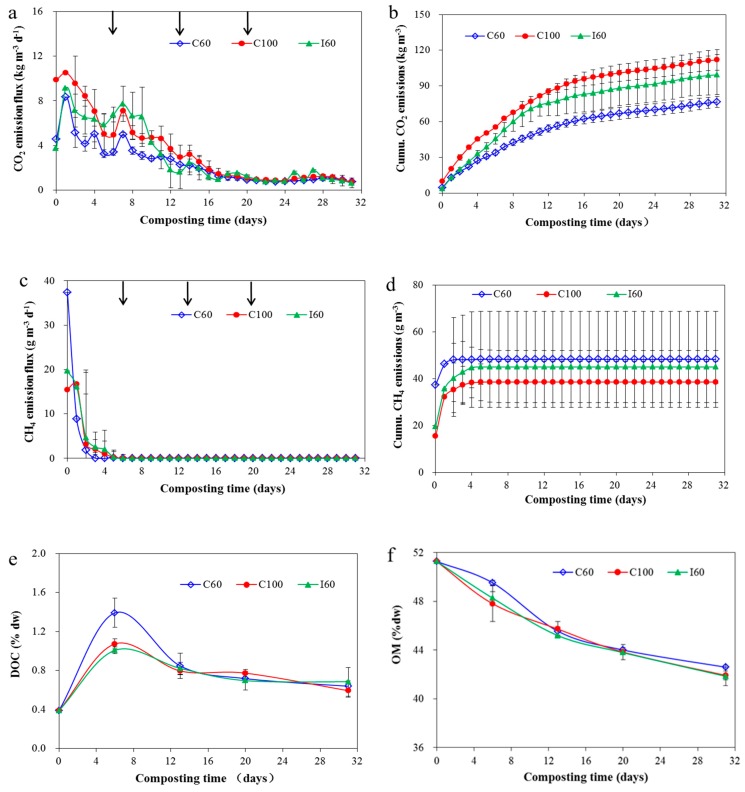
Change of CO_2_ (**a**), cumulative CO_2_ (**b**), change of CH_4_ (**c**), cumulative CH_4_ (**d**) emissions, change of DOC (**e**) and change of OM (**f**) during composting. Results are the mean of three replicates and error bars indicate standard deviation. Arrows indicate the time of turning. For abbreviations see Figure 1.

As the major form of carbon loss, CO_2_ emissions can clearly reflect the composting efficiency [43]. CO_2_ emissions correlated well with the composting temperature for the three treatments (r = 0.835–0.927, *p* < 0.001). The sharp decrease of CO_2_ on the second day was due to the inhibited microbial activity caused by excessively high temperatures [44]. The turning on the sixth day induced O_2_ to the compost pile, enabling aerobic microorganisms to decompose the OM and increase CO_2_ emissions accompanied with the increasing temperature in the following two days [45]. CO_2_ emissions then declined by the end of the thermophilic phase with the gradual consumption of the OM and decreasing temperature (Figure 3a and Figure 4a,f). Low CO_2_ emission was observed through the maturation in the curing phase, reflecting the stability of the mature compost [43].

The C100 group showed the highest cumulative CO_2_ emissions, with the highest daily CO_2_ emission flux mostly occurring during the initial period (Figure 4a,b). However, high CO_2_ emissions of the I60 group occurred during the period between the first and second turns (Figure 4a). This phenomenon was due to the high temperature in I60 treatment (Figure 3a). The high temperature of the I60 group indicated high aerobic microbial activity, which contributed to high CO_2_ emissions. For the two groups with continuous aeration, the compost with higher aeration rate showed higher CO_2_ emissions, consistent with the results from other studies [46,47]. The higher CO_2_ emissions could be attributed to two aspects. The first aspect was the high flow rate, which helped to achieve a high diffusion effect in the pile and expel CO_2_ into the air [12]. The second aspect was the high O_2_ for aerobic microorganisms that emanated CO_2_ emissions at suitable OM content and temperature [46].

CH_4_ emissions occurred during the initial composting stage and then ceased in a few days (Figure 4c). This emission tendency was comparable with CH_4_ emissions from previous reports by Wang et al. [34] and Jiang et al. [48] related to pig manure composting. The initially large CH_4_ emissions might be due to the presence of anaerobic microflora in the compost raw material because of the long ageing in the bedding house before experiment. Meanwhile, the settling of the compost matrix during the early phase of composting could have limited O_2_ diffusion, thereby forming a partially anaerobic environment required for CH_4_ emissions [46]. With the supply of fresh air into the composting reactor, O_2_ concentration began to increase [25] and CH_4_ emission decreased. Moreover, all CH_4_ emissions completely ceased after the first turn on the sixth day, an outcome that introduced O_2_. Among the three aeration treatments, the lowest cumulative CH_4_ emission was achieved in the C100 group (Figure 4d). The highest aeration rate aided to achieve better aerobic condition in the compost pile and resulted in the lowest CH_4_ emission [11,12,47].

CO_2_ and CH_4_ emissions during composting were mainly due to the decomposition of C-related matters, resulting in the continuous decrease of OM (Figure 4f). With prolonged time, the decreasing trend of OM gradually flattened, coinciding with the low daily C-related gas emissions. This tendency was highly consistent with those found in previous studies [5,49]. The lowest C-related gas emissions also resulted in the final highest OM content in C60 treatment. However, DOC content rapidly increased during the initial period, decreased, and then plateaued in the curing stage (Figure 4e). This phenomenon could also be found in Khan et al. [50] for pig manure composting. The increased DOC sourced from the released soluble organic matter was due to the decomposition of solid polymeric material in the composting matrix under high temperature and high MC during the initial phase [51]. DOC was the most active part in the compost material, and extremely high DOC content resulted in high CO_2_ emissions during the thermophilic phase.

For C balance, the TOC and CO_2_-C loss ratios in this study were comparable with the results of various studies on animal manure composting [12,52,53,54] (Table 2), in which a TOC loss ratio range of 46.6–58.5% and a CO_2_-C loss ratio range of 30.1–48.0% were reported. However, CH_4_-C loss ratios were in the lower range of the value of 0.01%–5% reported in other studies [12,34,52,54]. I60 showed the highest TOC loss ratio of 53.1%; this result was comparable with Jiang et al. [12], who also reported the intermittent aeration could achieve the highest TOC loss of 56.3%, which was higher than other continuous aeration strategies. The largest CO_2_ emissions occurred with the highest aeration rate in C100, as high aeration rate enhanced microbial activity by increasing O_2_ diffusion rate thus producing more CO_2_, and CO_2_ can always be expelled from the pile with continuous aeration [25,46]. The wide range of CH_4_-C loss in different studies revealed that the CH_4_ emission was an indicator which was relatively sensitive to the aeration condition. Forced aeration and small pile volume aided to maintain a good aerobic environment in the compost, thus resulting in low CH_4_-C losses in this study. Above all, C60 showed the lowest TOC and CO_2_-C losses, which were significantly lower than those for C100 treatment (*p* < 0.05). Thus, C60 was recommended because it reduced C-related gas emissions and preserved substantial OM in the compost product.

### 3.3. Evaluation of N Transformation during Composting

**Figure 5:**
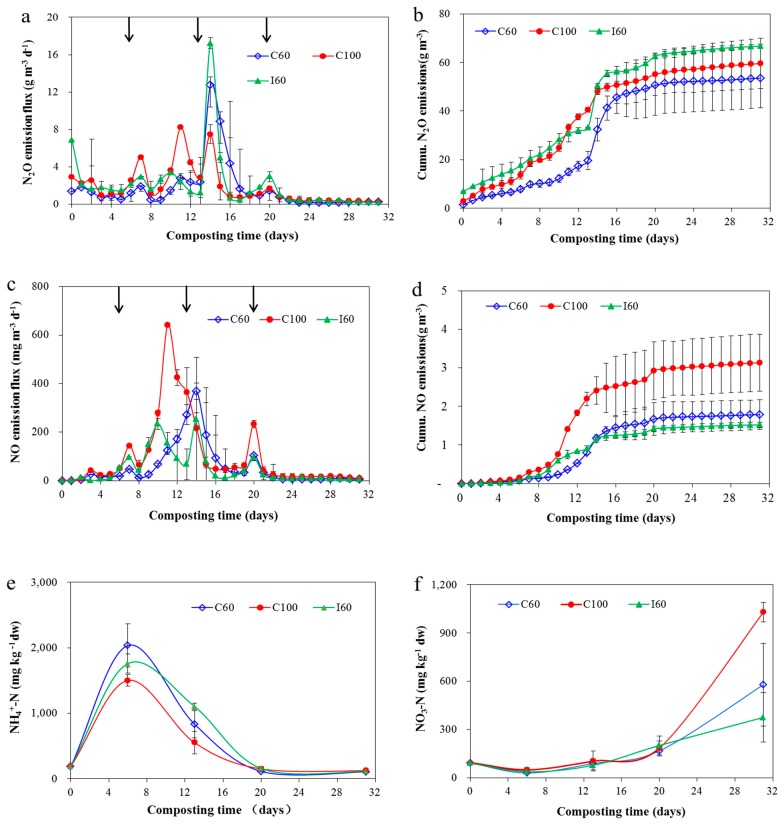
Change of N_2_O (**a**), cumulative N_2_O (**b**), change of NO (**c**), cumulative NO (**d**), emissions, change of NH_4_^+^-N (**e**) and change of NO_3_-N (**f**) during composting. Results are the mean of three replicates and error bars indicate standard deviation. Arrows indicate the time of turning. For abbreviations see Figure 1.

N_2_O emissions during composting followed a complex mechanism correlated to nitrification and denitrification. N_2_O emission was low during the initial phase of composting and increased after the first turn (Figure 5a). The N_2_O emission pattern corresponded well with that of Jiang et al. [48], who recorded N_2_O emissions for a specific period during manure composting. Usually, composting heap can form a heterogeneous environment in which aerobic zones with nitrification and anaerobic zones with denitrification may exist simultaneously. N_2_O emissions didn’t occur during the thermophilic phase. High temperature and high free ammonia content inhibited the activity of nitrobacteria, which maintained the NO_3_^−^-N content at a low level during the thermophilic phase (Figure 5f) [48,55]. These conditions could not support nitrification or denitrification, resulting in no N_2_O emissions during the initial phase. After the first turn in the sixth day, a first small peak of N_2_O emission occurred along with the first NO emission peak (Figure 5c). The turning event supplied O_2_ to eliminate the anaerobic pockets between the composting matrix and formed a favorable condition for nitrification [43], resulting in N_2_O and NO emissions and the oxidation of NH_4_^+^-N to NO_3_^−^-N (Figure 5e,f). During composting, large N_2_O emissions occurred during the mesophilic stage after the second turn, in which the inhibition effect was relieved when the temperature began to drop below 50 °C [56]. Among the three treatments, intermittent aeration treatment showed the highest cumulative N_2_O emissions because of the sharp increase of N_2_O after the second turn. Intermittent aeration increased the nitrification/denitrification alternation and achieved the highest N_2_O production in I60 [12], while C100 emitted more N_2_O emissions than C60 because of the higher aeration rate. The same results were also reported by Jiang et al. [12] considering the effect of aeration strategy on N_2_O emissions, indicating that C60 would be more preferred when considering reducing N_2_O emissions.

Nitric oxide emission showed a good correlation with N_2_O emission (Figure 5a,c). Usually, NO is deemed a direct intermediate of the denitrification pathway. During denitrification, nitrite is reduced to NO by nitrite reductase, which is further reduced by nitric oxide reductase to N_2_O [57,58]. Meanwhile, NO is a by-product of the nitrification pathway and can be produced during nitrification. In the present study, NO emission peaks consistently occurred after the turning during the entire process, except for the extremely high NO emission peak before the second turn in C100 treatment. Turning may increase NO emissions due to three aspects. First, the NO produced by denitrification process in the anaerobic zone may be moved to the surface area, thus increasing its diffusion potential; then, turning helped to redistribute the raw materials used for nitrification or denitrifaction; for instance, turning moved nitrate-rich material from top to bottom while moving NH_4_^+^ rich material from bottom to top, thus providing the material for nitrification on the surface while stimulating denitrification in the bottom [9]; third, the compaction condition was relieved after turn, and the air circulation would be optimized thus leading the increase of all gases [59]. Besides, aeration would also have some influences on NO emissions sourcing from nitrification process. C100 treatment had the highest aeration rate, indicating that sufficient O_2_ was supplied through aeration in this treatment and good aeration condition was suitable for nitrification. Consequently, the peak emission of NO was achieved and its second peak was prevented from synchronously occurring with the second turning. C100 showed the highest cumulative NO emissions of 3.1 ± 0.7 g·m^−3^, while intermittent aeration treatment I60 showed the highest cumulative N_2_O emissions of 66.7 ± 0.7 g·m^−3^; and C60 and I60 had similar cumulative NO emissions being of 1.5-1.8 g·m^−3^. Fukumoto et al. [19] reported the cumulative NO emission in a 90 days pig manure composting period with a working volume of 100 L was 62.4 g·m^−3^; however, there was almost no NO emission during the initial 30 days in Fukumoto et al., which was even lower than the NO emissions in this study. The complex mechanism related to nitrification and denitrification was the leading factor for the inconsistent emission tendency of N_2_O and NO for the three treatments, and further research is warranted to explain the more detailed mechanism from the view of microbiological analysis.

NH_4_^+^-N first showed an increasing trend followed by a rapidly decreasing pattern, and NO_3_^−^-N rapidly increased in the later composting stage (Figure 5e,f). N_2_O could be an intermediate product of denitrification [60]. NO_3_^−^-N in aerobic zones may move to anaerobic zones by diffusion or mass flow, and then NO_3_^−^-N was denitrified to N_2_O and N_2_ in the anaerobic zones. The transformation contributed to the small accumulation of NO_3_^−^-N during this phase before the third turning and partially resulted in the decreasing trend of NH_4_^+^-N, which was continuously transformed to NO_3_^−^-N during nitrification. During the curing phase after the third turn, NO_3_^−^-N sharply accumulated, and N_2_O or NO emission was not detected in this phase. The pile temperature in this stage was approximately the same as the ambient air temperature at 30 °C, and the rate of denitrification decreased with low temperature [61], resulting in the accumulation of NO_3_^−^-N in the pile rather than of N_2_O or NO emissions. C100 treatment showed the highest final NO_3_^−^-N content, indicating that high O_2_ supply supported nitrification and resulted in the highest transformation from NH_4_^+^-N to NO_3_^−^-N. On the contrary, I60 showed the lowest final NO_3_^−^-N content, which may be caused by the greatest conversion of NO_3_^−^-N to N_2_O emissions during the composting process in I60 [12].

For N balance, TN loss ranged from 29.8% to 35.9% during composting, N_2_O-N loss ranged from 0.74% to 0.93%, and NO-N loss ranged from 0.02% to 0.03% (Table 2). The loss ratios of TN and N_2_O in this study fell into the range of 11.4% to 73.8% and 0.02% to 2.02%, respectively, which were reported from the meta-analysis studies for evaluating N related losses from beef cattle manure composting, swine manure composting and chicken manure composting [62,63,64], but obviously our results in this study were in a relatively low range. This may be caused by the fact that usually TN content of beef cattle manure was lower than that of other animal manures [18,65], because of the difference in dietary composition, with usually higher crude protein content in chicken and swine feed than beef cattle feed. Moreover, the long storage period of manure in bedding house may also cause some easily degradable N such as NH_4_^+^-N being degraded or transformed to other stable forms, thus causing the low N loss during this composting period. Moral et al. [66] reported after cattle manure being stored for 52 days, total N mass reduced by 15.6%; however, the easily decomposable NH_4_^+^-N mass reduced by 77.1%. Few studies have been conducted on NO emissions from manure composting. Martins and Dewes [18] has reported NO_X_-N emissions lower than 5%, and Fukumoto et al. [19] reported NO-N loss was 0.83–3.92% of initial TN during swine manure composting, which seemed much higher than our result. However, the N_2_O-N loss was 5.6–6.5%, and NH_3_-N loss was 9.2–12.6% in Fukumoto et al. [19], which meant that the nitrification and denitrification effect was strong thus huge emission of N_2_O happened while less NH_3_ emission occurred than in the normal conditions. Therefore, the high N_2_O emissions meant high NO emissions would occur simultaneously in Fukumoto et al. [19]’s study, thus leading the much higher NO loss ratio than our result.

Generally, I60 had the highest TN and N_2_O-N losses, whereas C60 had opposite results, meaning that C60 would be more recommended considering preserving N in the final compost besides reducing N_2_O emissions. Meanwhile, unaccounted part of N in I60 was the highest among the three treatments. Sun [67] also found that TN and N_2_O-N losses were higher in intermittent aeration treatment than in continuous aeration. Usually, high NH_3_ emissions occurred in the compost, and a large ratio of unaccounted N loss in this study existed in the form of NH_3_. Meanwhile, high N_2_O emissions indicated the occurrence of nitrification and denitrification in I60 treatment; thus, end products, such as N_2_ would occupy some parts of unaccounted N [28]. Moral et al. [66] reported that N_2_-N emission was 5 times higher than NH_3_-N emission during cattle farmyard manure storage.

### 3.4. Evaluation of S Transformation during Composting

**Figure 6:**
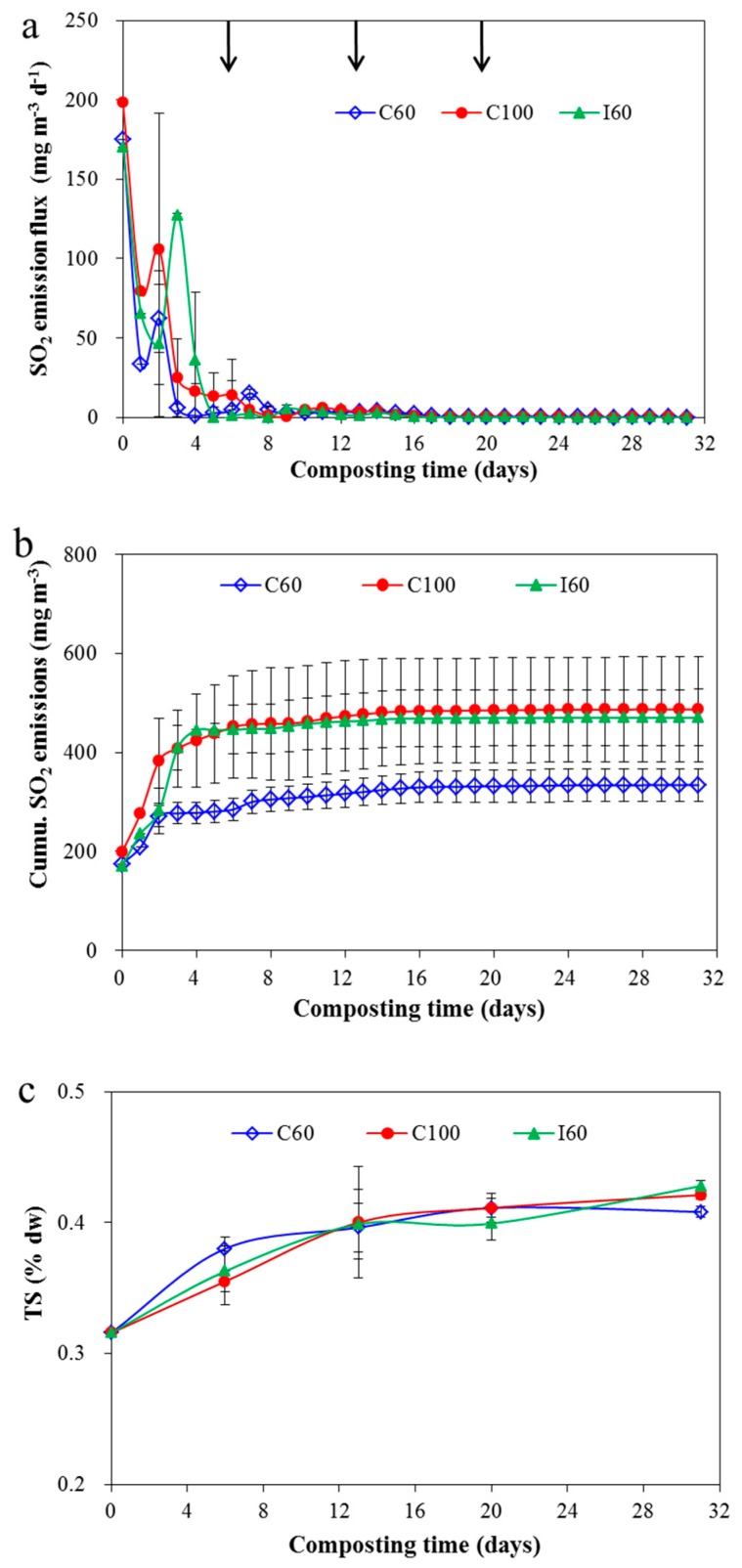
Change of SO_2_ (**a**), cumulative SO_2_ (**b**) emissions, change of TS (**c**) during composting. Results are the mean of three replicates and error bars indicate standard deviation. Arrows indicate the time of turning. For abbreviations see Figure 1.

S-related gas emissions were low during the composting period. H_2_S emission was not detected in the composting period, while only some SO_2_ emissions occurred during the initial period, and no emission occurred after the 10th day (Figure 6). During the early phase of composting, aerobic condition performed poorly and some microorganisms decomposed the compost material to SO_2_ as incomplete oxidation products. Ni et al. [20] observed the SO_2_ emission from pig manure storage, and also reported that the highest releases of SO_2_ being 25–168 μg·h^−1^ occurred during the first week of the one month storage, and then it decreased to a quite low level in the middle of the test, but finally came up and remained at about 30 μg·h^−1^ during the last nine days. H_2_S was produced in an anaerobic condition and usually it could be observed during the initial phase of composting [68]. However, no H_2_S was detected during the entire composting in this study. H_2_S was an acidic gas, thus the more alkaline initial pH was the leading factor for the nil emission of H_2_S. Arogo et al. [69] reported that maintaining manure pH > 7 could reduce H_2_S release by keeping H_2_S mostly in the ionic forms. In addition, even the H_2_S produced, it would be easily absorbed by the composting material because of the high pH.

TS concentration increased by 29%–36% at the end of composting because of the concentration effect (Figure 6c). For S balance, total S loss ranged from 19.6% to 21.9% during composting (Table 2), which fell in the range of 2.4%–28.7% of TS loss for pig manure composting or kitchen waste composting [15,17,27,67]. Low SO_2_ loss and no H_2_S loss resulted in a high unaccounted part of S loss of 17.5%–19.9%. In addition to SO_2_ and H_2_S emissions, many other VSC-related emissions, such as dimethyl disulfide (Me_2_SS), dimethyl sulfide (Me_2_S), and methyl mercaptan, occurred during composting. These parts of S-related gas emissions would even be much higher than those of H_2_S emissions [15,17,27,68]. For example, Zang et al. [15] reported a TS loss of 18.3% for pig manure composting. Among the losses, H_2_S-S loss was lower than 0.001%, while the Me_2_S and Me_2_SS were 2.2% and 4.4%, respectively, and the unaccounted part of S was 11.5%. Zang et al. [27] obtained a high Me_2_SS-S loss of 6.0% during pig manure composting.

### 3.5. GHG Emissions during Composting

The GHG emission of C60 was 119 ± 28 kg CO_2_eq·t^−1^ DM, which was the lowest among the three strategies (Table 3). In this study, the contribution ratios of N_2_O to GHG were high, ranging from 46% to 60%. Jiang et al. [12] reported N_2_O was the predominant GHG from all emissions, accounting for 42–72% of the total GHG emissions, which was comparable with our results. Meanwhile, energy consumption was also quite important with the contribution ratio of 36–51% observed in this study. It can be calculated that if only the CH_4_ and N_2_O emissions were considered, the GHG emissions from C100 were lower than that from I60; however, when energy consumption was taken into account, C100 produced more GHG than I60, indicating the importance of energy consumption for GHG emission. Pan et al. [40] reported GHG emissions of approximately 34–104 kg CO_2_eq·t^−1^ DM from the compost of sewage sludge. Li et al. [52] reported GHG emissions of 138 kg CO_2_eq·t^−1^ DM during pig manure composting with the addition of spent mushroom substrates. In these two indicated studies, N_2_O was also defined as the major GHG contributor, with its ratio ranging from 62.8% to 98.0% [40,52]; however, the GHG sourcing from energy consumption was not included, causing the contribution ratio of N_2_O being higher than that in this study. Thus, C60 would be recommended when considering reducing energy consumption and GHG emissions.

## 4. Conclusions

Aeration strategy had a strong influence on C, N, and S transformation during manure composting. Intermittent aeration increased the alternation of nitrification and denitrification, and led to the highest N_2_O emissions; meanwhile the TOC and TN loss were also the highest in I60 among the three aeration strategies. C60 showed an overall opposite effects. For N elements, C60 had the lowest N_2_O emissions and TN loss; for C elements, C60 achieved the lowest CO_2_ emissions and TOC loss; meanwhile, the GHG emissions in C60 were also the lowest because of the lowest N_2_O emissions and the lower energy consumption, as these two parts contributed nearly 96% of the total GHG emissions. Thus C60 aeration strategy was recommended for cattle manure composting when considering both elements conservation and GHG mitigation.

## Figures and Tables

**Table 1:** Physical and chemical properties of raw materials and mixture (values are means ± SD, *n* = 3) ^a^.

Parameters	TOC% dw	TN% dw	TS% dw	MC%	C/N	pH (solid:water = 1:10)
Cattle manure	20.6 ± 0.6	2.44 ± 0.35	0.38 ± 0.01	69.8 ± 3.1	8.4 ± 0.21	9.0 ± 0.0
Wheat straw	31.2 ± 0.7	0.79 ± 0.09	ND	8 ± 0.26	39.4 ± 0.43	ND
Compost mixture	29.7 ± 0.4	2.10 ± 0.23	0.32 ± 0.01	65.4 ± 0.5	14.2 ± 0.26	9.1 ± 0.0

^a^ TOC (total organic carbon), TN (total nitrogen), TS (total sulfur), MC (moisture content), dw (dry weight), ND (not detected).

**Table 2:** C, N and S balance (values are means ± SD, *n* = 3) ^a^.

Treatment	CH_4_-C Loss (% Initial TC)	CO_2_-C Loss (% Initial TC)	TOC Loss (% initial TOC)	N_2_O-N Loss (% Initial TN)	NO-N Loss (% Initial TN)	TN Loss (% Initial TN)	SO_2_-S Loss (% Initial TS)	TS Loss (% Initial TS)
C60 ^b^	0.05 ± 0.04	31.5 ± 3.3 ^B^	48.8 ± 1.7 ^B^	0.74 ± 0.30	0.02 ± 0.01	29.8 ± 2.5 ^B^	0.02 ± 0.00	20.3 ± 4.4
C100	0.04 ± 0.02	46.1 ± 6.4 ^A^	50.7 ± 0.6 ^A,B^	0.83 ± 0.25	0.03 ± 0.01	31.0 ± 0.5 ^B^	0.03 ± 0.01	19.6 ± 1.3
I60	0.05 ± 0.01	40.9 ± 10.2 ^A,B^	53.1 ± 2.2 ^A^	0.93 ± 0.01	0.02 ± 0.00	35.9 ± 3.3 ^A^	0.03 ± 0.01	21.9 ± 6.8

^a^ Means within each column followed by different letters are significantly different (*p* < 0.05); ^b^ For abbreviations see Figure 1.

**Table 3:** GHG emissions and relative contributions of each sector from manure composting (values are means ± SD, *n* = 3) ^a^.

Treatment	Power ^b^	CH_4_	N_2_O	GHG
	kg CO_2_eq·t^−1^ DM ^c^ (%)			kg CO_2_eq·t^−1^ DM
C60 ^d^	47.9 ± 0 (40) ^B^	6.1 ± 4.5 (5)	64.9 ± 25.8 (55)	118.8 ± 27.8
C100	79.8 ± 0 (51) ^A^	4.8 ± 1.9 (3)	72.1 ± 22.0 (46)	156.7 ± 20.8
I60	47.9 ± 0 (36) ^B^	5.7 ± 1.3 (4)	80.8 ± 1.1 (60)	134.3 ± 2.3

^a^ Means within each column followed by different letters are significantly different (p < 0.05); ^b^ Aeration power calculation based on: 1 m^3^ aeration (in standard condition) needs 0.004 kWh power consumption, and coal-fired power station data that 1 kWh power production emits 0.997 kg CO_2_ (Jiang et al., 2015); ^c^ DM = dry matter; ^d^ For abbreviations see Figure 1.
